# An astonishing wealth of new proteasome homologs

**DOI:** 10.1093/bioinformatics/btab558

**Published:** 2021-07-29

**Authors:** Adrian C D Fuchs, Vikram Alva, Andrei N Lupas

**Affiliations:** Department of Protein Evolution, Max Planck Institute for Developmental Biology, 72076 Tübingen, Germany; Department of Protein Evolution, Max Planck Institute for Developmental Biology, 72076 Tübingen, Germany; Department of Protein Evolution, Max Planck Institute for Developmental Biology, 72076 Tübingen, Germany

## Abstract

**Motivation:**

The proteasome is the main proteolytic machine for targeted protein degradation in archaea and eukaryotes. While some bacteria also possess the proteasome, most of them contain a simpler and more specialized homolog, the heat shock locus V protease. In recent years, three further homologs of the proteasome core subunits have been characterized in prokaryotes: Anbu, BPH and connectase. With the inclusion of these members, the family of proteasome-like proteins now exhibits a range of architectural and functional forms, from the canonical proteasome, a barrel-shaped protease without pronounced intrinsic substrate specificity, to the monomeric connectase, a highly specific protein ligase.

**Results:**

We employed systematic sequence searches to show that we have only seen the tip of the iceberg so far and that beyond the hitherto known proteasome homologs lies a wealth of distantly related, uncharacterized homologs. We describe a total of 22 novel proteasome homologs in bacteria and archaea. Using sequence and structure analysis, we analyze their evolutionary history and assess structural differences that may modulate their function. With this initial description, we aim to stimulate the experimental investigation of these novel proteasome-like family members.

**Availability and implementation:**

The protein sequences in this study are searchable in the MPI Bioinformatics Toolkit (https://toolkit.tuebingen.mpg.de) with ProtBLAST/PSI-BLAST and with HHpred (database ‘proteasome_homologs’). The following data are available at https://data.mendeley.com/datasets/t48yhff7hs/3: (i) sequence alignments for each proteasome-like homolog, (ii) the coordinates for their structural models and (iii) a cluster-map file, which can be navigated interactively in CLANS and gives direct access to all the sequences in this study.

**Supplementary information:**

Supplementary data are available at *Bioinformatics* online.

## 1 Introduction

The proteasome is the main cytosolic protease complex in eukaryotes, archaea and some bacteria. It is a barrel-shaped particle, often referred to as the 20S proteasome, which interacts with various regulators or unfoldases that modulate its function (Forouzan *et al.*, 2012; Fort *et al.*, 2015; Muller and Weber-Ban, 2019). For instance, in the eukaryotic targeted protein degradation pathway, it is capped by the 19S regulatory particle, a AAA+ (ATPases associated with diverse cellular activities)-containing complex, to form the 26S complex (Collins and Goldberg, 2017). To be degraded, proteins are tagged with ubiquitin, a small regulatory protein recognized by the 19S particle. The ubiquitin tag is subsequently removed, and the target protein is unfolded and threaded through the proteolytic channel of the proteasome for degradation. Therefore, this system is of high physiological and pharmaceutical importance, but its study is complicated due to its immense complexity (Thibaudeau and Smith, 2019). Over the years, it has become increasingly evident that many of its components do not serve just a single purpose, but have inherited many less understood functions and characteristics from their prokaryotic progenitors (Maupin-Furlow, 2011; Nunoura *et al.*, 2011; Zaremba-Niedzwiedzka *et al.*, 2017). This is because the proteasome and its accessory proteins must have evolved independently before they developed the ability to co-operate in the modern ubiquitin-proteasome system. For instance, ubiquitin and some of its modifiers evolved from a cofactor synthesis pathway (Burroughs *et al.*, 2009; 2012; Fuchs *et al.*, 2018b; Hennell James *et al.*, 2017; Hepowit *et al.*, 2012; Humbard *et al.*, 2010; Maupin-Furlow, 2011), and many proteasomal unfoldases also serve as chaperones that assist in protein folding (Benaroudj and Goldberg, 2000; Forouzan *et al.*, 2012). Likewise, even the uncapped 20S proteasome, a form of the proteasome free of its interactors, appears to have additional functions, such as the clearance of oxidized, toxic or unstructured proteins (Kumar Deshmukh *et al.*, 2019; Olshina *et al.*, 2020; Raynes *et al.*, 2016; Sahu *et al.*, 2019). Consequently, the study of simpler prokaryotic homologs is not only of interest for our understanding of prokaryotic physiology, but their characterization also helps us understand the origins of the eukaryotic system and why it works as it does.

While heat shock locus V (HslV) (Rohrwild *et al.*, 1996), which is found in bacteria and some eukaryotic organelles of endosymbiotic origin, was the only known proteasome homolog for many years, we and others have recently characterized three further proteasome homologs: the bacterial complexes Anbu (Fuchs *et al.*, 2017; Gille *et al.*, 2003; Piasecka *et al.*, 2018; Valas and Bourne, 2008; Vielberg *et al.*, 2018) and BPH (Fuchs *et al.*, 2018a), and the monomeric archaeal connectase (Fuchs *et al.*, 2021). In the Pfam database (Finn *et al.*, 2014), the proteasome subunits, HslV, BPH and Anbu are classified into the ‘Proteasome subunit’ family within the ‘NTN hydrolase’ clan (N-terminal nucleophile). Given the increasing number of homologous proteins, we find it more appropriate to refer to this family as the proteasome-like family in the following. We wish to emphasize that ‘proteasome-like’ does not imply the capability to self-assemble or to hydrolyze polypeptides, but rather evolutionary relatedness.

**Fig. 1.**
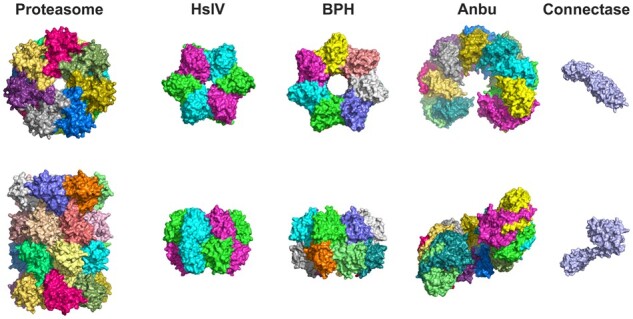
Structural diversity of proteasome-like homologs. Shown are top and side views of the proteasome (PDB code: 1PMA), and its homologs HslV (1G3K), BPH (5OVS), Anbu (5LOX) and connectase (6ZVZ)

Despite being built of structurally similar subunits with a conserved catalytic center, these proteins exhibit distinct quaternary structures (Fig. 1). The proteasome consists of four axially stacked heptameric rings, of which the two outer rings are formed by the enzymatically inactive α subunits and the two inner rings by the catalytic β subunits. In eukaryotes, these rings consist of 14 different but homologous subunits, seven of the α-type and seven of the β-type, whereas in prokaryotes, they are built from one or a few subunits of each type. HslV and BPH each consist of just two homo-oligomeric rings of proteasome β-like subunits, hexameric in case of HslV and heptameric in that of BPH. The quaternary structures of the remaining two proteasome-like homologs are even more distinct: Anbu exhibits an open split-ring architecture, whereas connectase is a monomer. These different architectures also come with different functions.

While HslV interacts with the AAA+ unfoldase HslU and degrades proteins in a similar manner as the proteasome-AAA+ complex (Ramachandran *et al.*, 2002), no interacting unfoldases are known for BPH. We have previously proposed that BPH may instead have functions similar to those of the uncapped proteasome (Fuchs *et al.*, 2018a). Likewise, no function is known for the Anbu complex, but its unique split-ring architecture suggests a different mode of action (Fuchs *et al.*, 2017; Piasecka *et al.*, 2018; Vielberg *et al.*, 2018). Lastly, unlike the other proteasome-like homologs, connectase is not a protease, but instead acts as a highly specific protein ligase (Fuchs *et al.*, 2021).

**Fig. 2.**
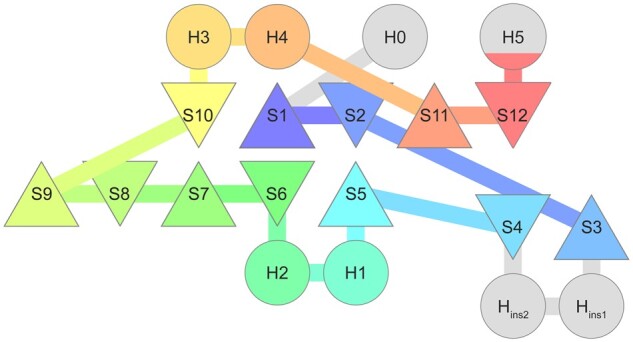
Architecture of the proteasome β subunit and its homologs. The secondary structure elements of the archetypical proteasome β subunit are colored, and insertions or extensions found in other homologs are shown in gray [i.e. H0 (proteasome α), H_ins1_/H_ins2_ (connectase), H5 (Anbu)]. Helices (H) are depicted as circles and sheets (S) as triangles. H1/H2 are not found in connectase and S12 is not found in connectase, BPH and HslV

On the subunit level, these structure-function relationships are typically mediated by homolog-specific α-helical insertions and extensions within the shared proteasome β-like fold (Fig. 2). In the archetypical proteasome β subunit as well as in individual subunits of HslV and BPH, the two central β-sheets (i.e. S1–S2/S10–S11 and S5–S9) harbor the catalytic center, while the four flanking helices (H1–H4) are involved in inter-subunit contacts (Lowe *et al.*, 1995). At their N-terminus, proteasome α subunits have an additional helical extension that enables them to regulate substrate access to the proteolytic channel. By contrast, monomeric connectase does not possess the first two helices (H1–H2) and instead uses a two-helical insertion (H_ins1_/H_ins2_) at a different position to control substrate specificity (Fuchs *et al.*, 2021). Finally, Anbu features a C-terminal helical extension (H5) that mediates a coiled-coil interaction between the two ring-layers and thereby stabilizes the split-ring architecture (Fuchs *et al.*, 2017; Piasecka *et al.*, 2018; Vielberg *et al.*, 2018).

Given the diversity and central physiological role of some of these proteins, it appears likely that they emerged quite early in evolution. The proteasome α and β subunits, for example, originated by gene duplication in an event that probably took place before the split between bacteria and archaea (Fuchs *et al.*, 2017; Zwickl *et al.*, 1992). Furthermore, it appears possible that the first representatives of this family acted in smaller assemblies or even as monomers and only later acquired the ability to form ring-shaped structures (Fuchs and Hartmann, 2019). Accordingly, other members of the clan encompassing the proteasome, the Ntn hydrolases, do not form these ring structures (Oinonen and Rouvinen, 2000), and even the proteasome-like family members Anbu and connectase do not form such rings.

To understand the evolutionary processes that led to the observed diversity, we conducted in-depth sequence analyses of all proteasome-like family members. To our surprise, we discovered a wealth of other yet unknown proteasome-like homologs. In the following, we describe these new representatives, their shared and unique features, and discuss their evolutionary relationships.

## 2 Materials and methods

### 2.1 Identification of proteasome-like homologs

All sequence searches were conducted with the PSI-BLAST module in the MPI Bioinformatics Toolkit (Alva *et al.*, 2016; Zimmermann *et al.*, 2018), using the August 2020 release of the NCBI non-redundant sequence database filtered for a maximum pairwise sequence identity of 70% (nr70). The E-value cutoff was set to 1, and all sequences were manually analyzed after each iteration. We started individual searches with proteasome subunits from *Saccharomyces cerevisiae*, *Thermoplasma acidophilum* and *Mycobacterium tuberculosis*, *Methanosarcina mazei* connectase, *Pseudomonas aeruginosa* Anbu, *Haemophilus influenzae* HslV and *Cupriavidus metallidurans* BPH. We then pooled all these sequences, removed multiple or partial sequences, and reviewed the multiple sequence alignments to ensure that they contained only proteasome-like homologs. This was typically ensured through the identification of the conserved active site residues and, in case of doubt, also through structure prediction. We then clustered all candidates using CLANS (Frickey and Lupas, 2004) with default parameters. In the resulting map, we identified the clusters around the above seed sequences, ‘new’ clusters that could not be traced to one of the seed sequences, and outlier sequences that were not part of these clusters at a *P*-value of 1e-10, i.e. formed few or no connections to other proteins at this *P*-value cutoff. The new clusters and the outlier sequences were then used individually for subsequent searches that were performed as described above. The hits from these searches were then pooled with the sequences included in the first cluster map, filtered as described above, and used for the generation of a second cluster map. This process was repeated, and in each round, the cluster map grew until no new sequences could be identified with this technique. In the final map, we identified new proteasome-like homologs as clusters that formed at a *P*-value of 1e-10, localized separate from other clusters, and contained at least 10 representatives. Finally, we gathered all non-redundant representatives of each cluster by performing PSI-BLAST searches against the August 2020 release of the non-redundant protein database.

### 2.2 Generation of the cluster map

**Fig. 3.**
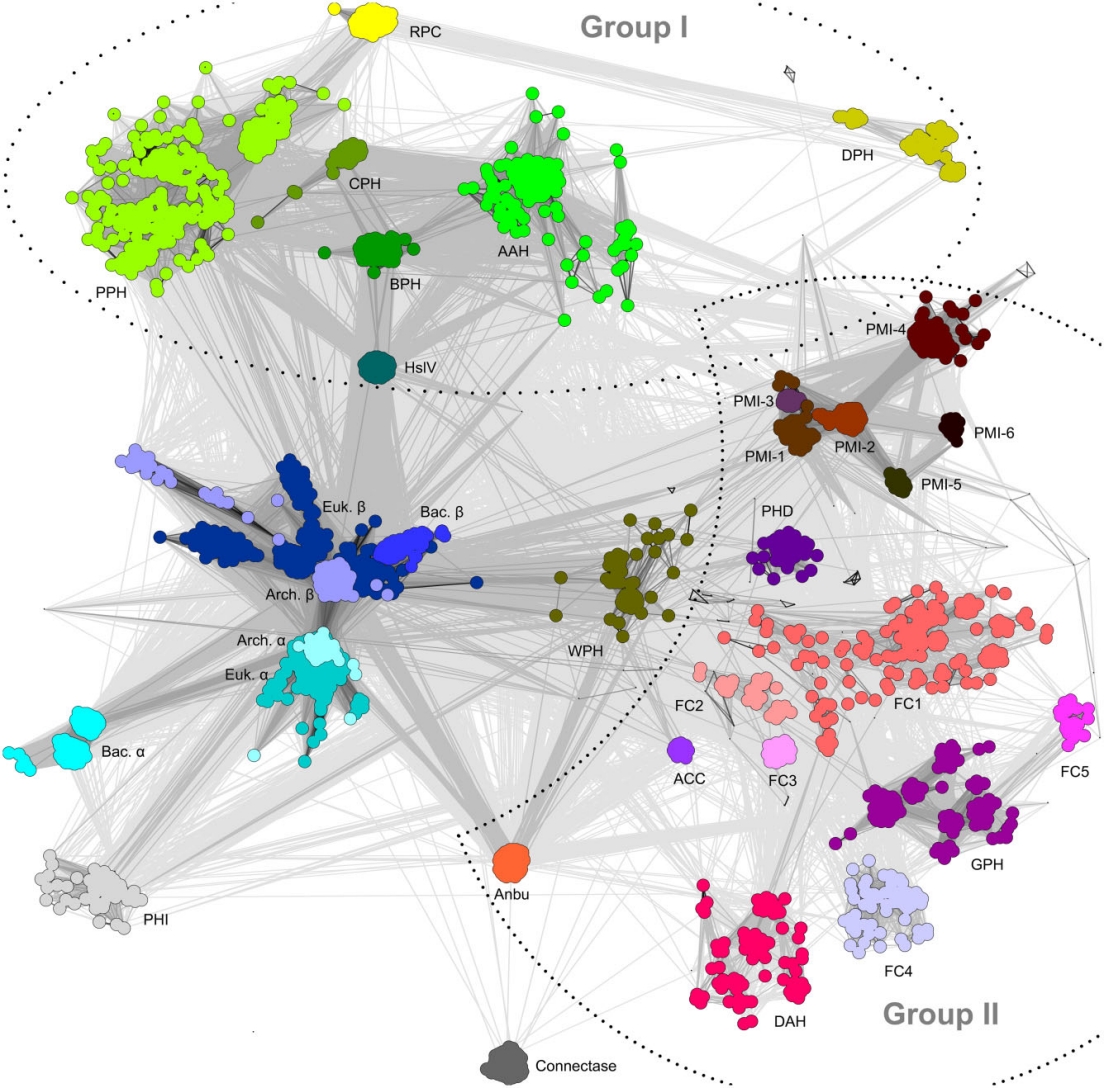
Cluster map of previously known and new members of the proteasome family. Shown is a representative set of 4821 protein sequences, symbolized as dots. Line coloring reflects the degree of their pairwise sequence similarities as measured by BLAST+ *P*-values; the darker a line, the higher the similarity. Sequence clusters represent individual proteasome-like family members and were colored accordingly. New proteasome-like homologs were named as described in the main text; archaeal (Arch.), bacterial (Bac.) and eukaryotic (Euk.) proteasome subunit (α, β) sequences are depicted in different colors. Clustering was performed at a *P*-value cutoff of 1e-3 and connections are shown at a *P*-value cutoff of 1e-2

For the generation of the cluster map shown in Figure 3, we filtered the sequences of each proteasome-like homolog to a maximum pairwise identity of 90% using MMseqs2, with the minimum alignment coverage set to 0.7 (Steinegger and Söding, 2017); Exceptions were the proteasome-like homologs with a high number of representatives, i.e. the proteasome α and β subunits, HslV, Anbu and PMI-4, which were filtered to 70% instead. This step greatly reduced the required calculation time without having noticeable effects on the resolution of the cluster map. The sequences were then used to calculate pairwise BLAST+ similarities with CLANS (Frickey and Lupas, 2004) using standard parameters. Clustering was performed at a *P*-value cutoff of 1e-3 and connections in the map are shown at a *P*-value cutoff of 1e-2. The map file is available for download at https://data.mendeley.com/datasets/t48yhff7hs/3.

### 2.3 HMM-HMM comparisons

**Fig. 4.**
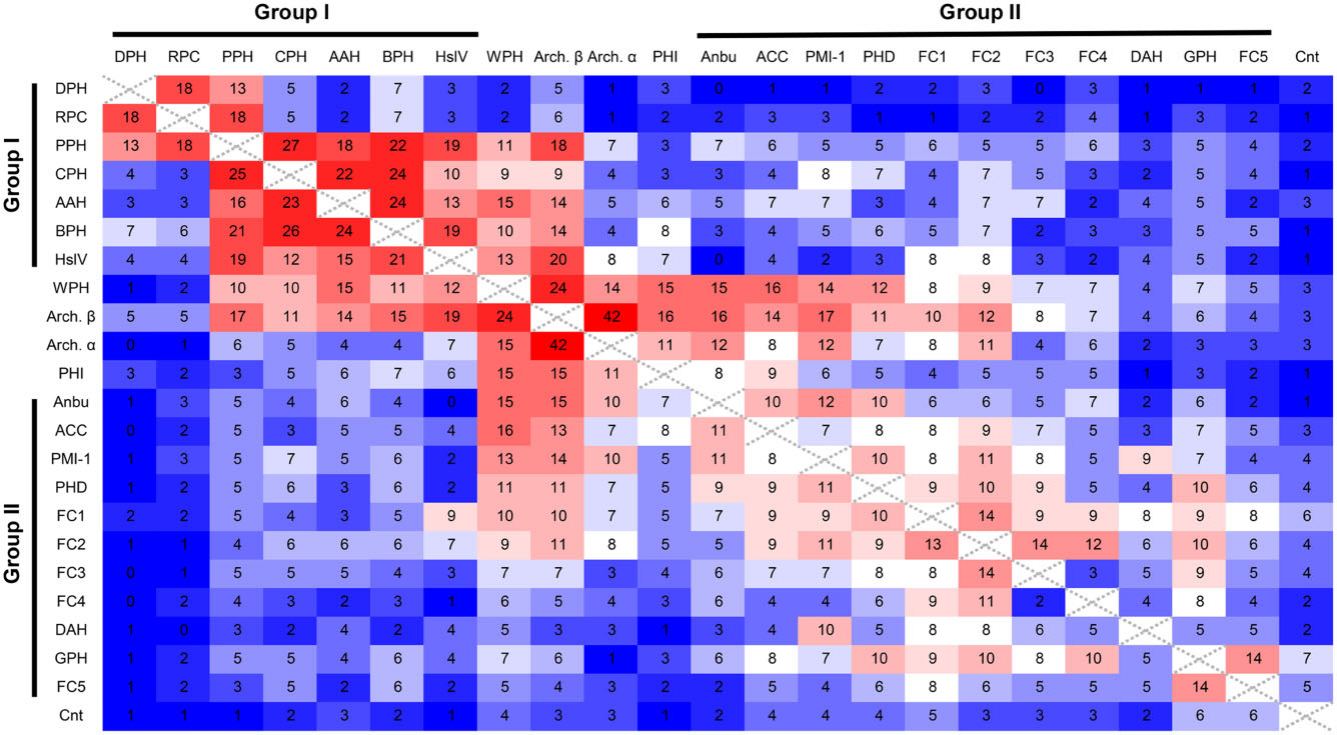
Pairwise HMM-HMM comparison of proteasome-like homologs. Profile HMMs were generated for each proteasome-like homolog based on multiple sequence alignments, and pairwise HMM-HMM comparisons were performed with HHpred (Zimmermann *et al.*, 2018). The numbers in the cells denote the negative E-value exponents yielded by HHpred as a measure of similarity. Highly similar protein pairs are colored red and less similar pairs blue. Connectase is abbreviated as Cnt

For HMM-HMM comparisons (Fig. 4), we filtered the sequences of each proteasome-like homolog to a maximum pairwise identity of 90% using MMseqs2, with minimum alignment coverage set to 0.7 (Steinegger and Söding, 2017). The sequences were then aligned with MUSCLE (Edgar, 2004), and the alignments were compared with HHpred (Soding, 2005). The heatmap shows the negative E-value exponent yielded by HHpred as a measure for the similarity of each protein pair.

### 2.4 Structure prediction

**Fig. 5.**
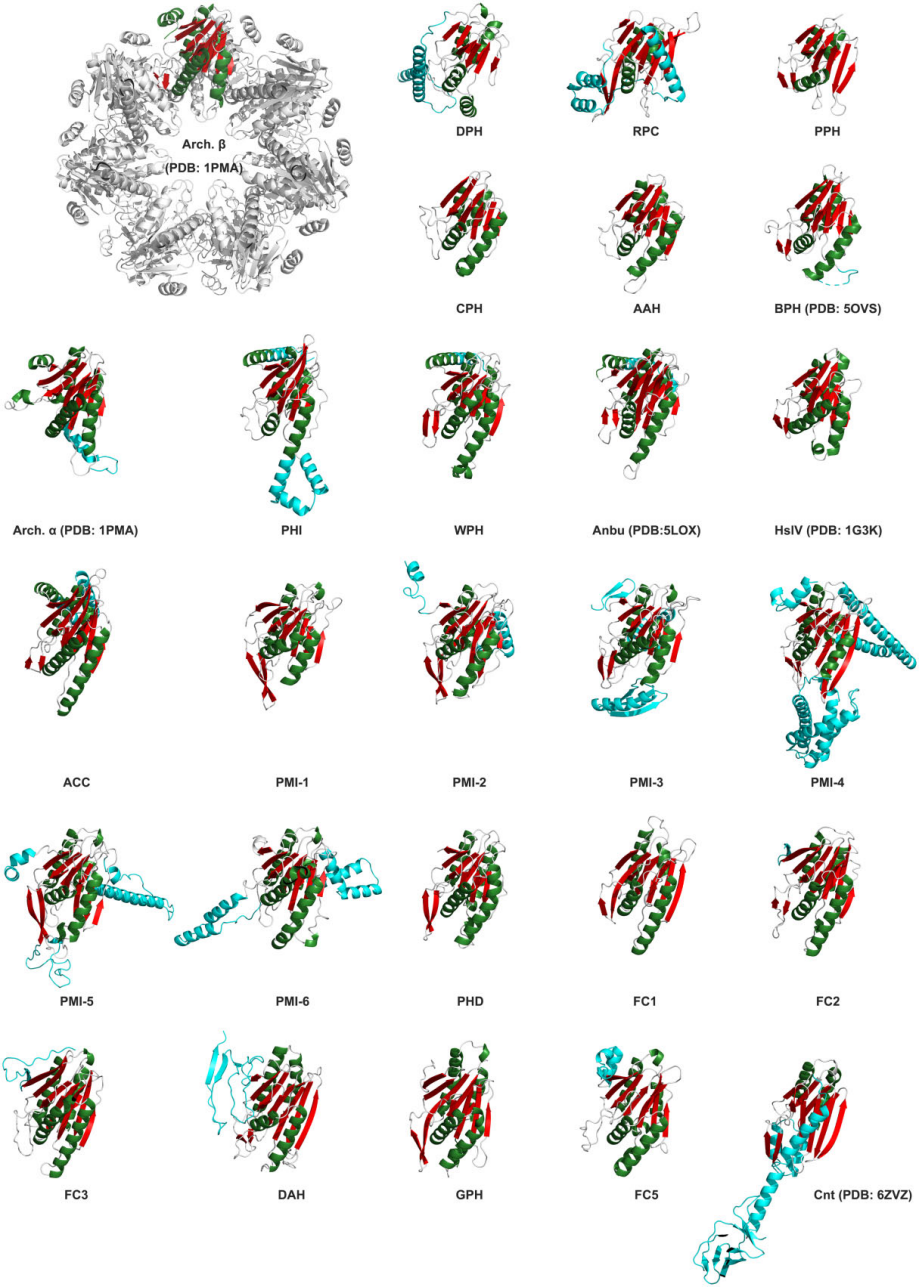
Subunit structures and models of proteasome-like homologs. Shown are structures of archaeal proteasome α and β (PDB code: 1PMA), BPH (5OVS), HslV (1G3K), connectase (6ZVZ) and Anbu (5LOX) subunits, as well as models of the other proteasome-like homologs. A representation of the heptameric proteasome β ring layer (top left) visualizes the orientation of these subunits and what their unique features could mean in the context of an assembled complex. Within the shared core, β-sheets are colored red and α-helices green, whereas protein-specific elements (see Fig. 6) are shown in cyan. No structure is shown for FC4 as we had concerns about the correctness of the predicted model. The models are available online at https://data.mendeley.com/drafts/t48yhff7hs/3. A model quality estimation using QMEANDisCo (Studer *et al.*, 2020) is shown in Supplementary Table S1

For structure prediction (Fig. 5), we employed the tFold server (https://drug.ai.tencent.com/console/en/tfold). As input, we used one representative sequence for each proteasome-like homolog. For the selection of these representative sequences, we created consensus sequences for each proteasome-like homolog and used them to detect the most similar natural sequence. The accession numbers of these sequences are: WP_074989469.1 (DPH), WP_140207863 (RPC), WP_042125023.1 (PPH), WP_008185288 (CPH), WP_035979212 (AAH), WP_012956871.1 (PHI), WP_040339656.1 (WPH), WP_161937350.1 (ACC), WP_115649681.1 (PMI-1), WP_137109689.1 (PMI-2), OGZ26396.1 (PMI-3), HDY66466.1 (PMI-4), WP_179179973.1 (PMI-5), WP_103790100.1 (PMI-6), OGP72033.1 (PHD), WP_075525441.1 (FC1), WP_000217171.1 (FC2), WP_098409529.1 (FC3), WP_076223917.1 (FC4), WP_085249540.1 (DAH), WP_152342571.1 (GPH) and WP_106036939.1 (FC5). All models are available for download at https://data.mendeley.com/datasets/t48yhff7hs/3. The model quality was estimated using QMEANDisCo (Studer *et al.*, 2020).

### 2.5 Sequence alignment

**Fig. 6.**
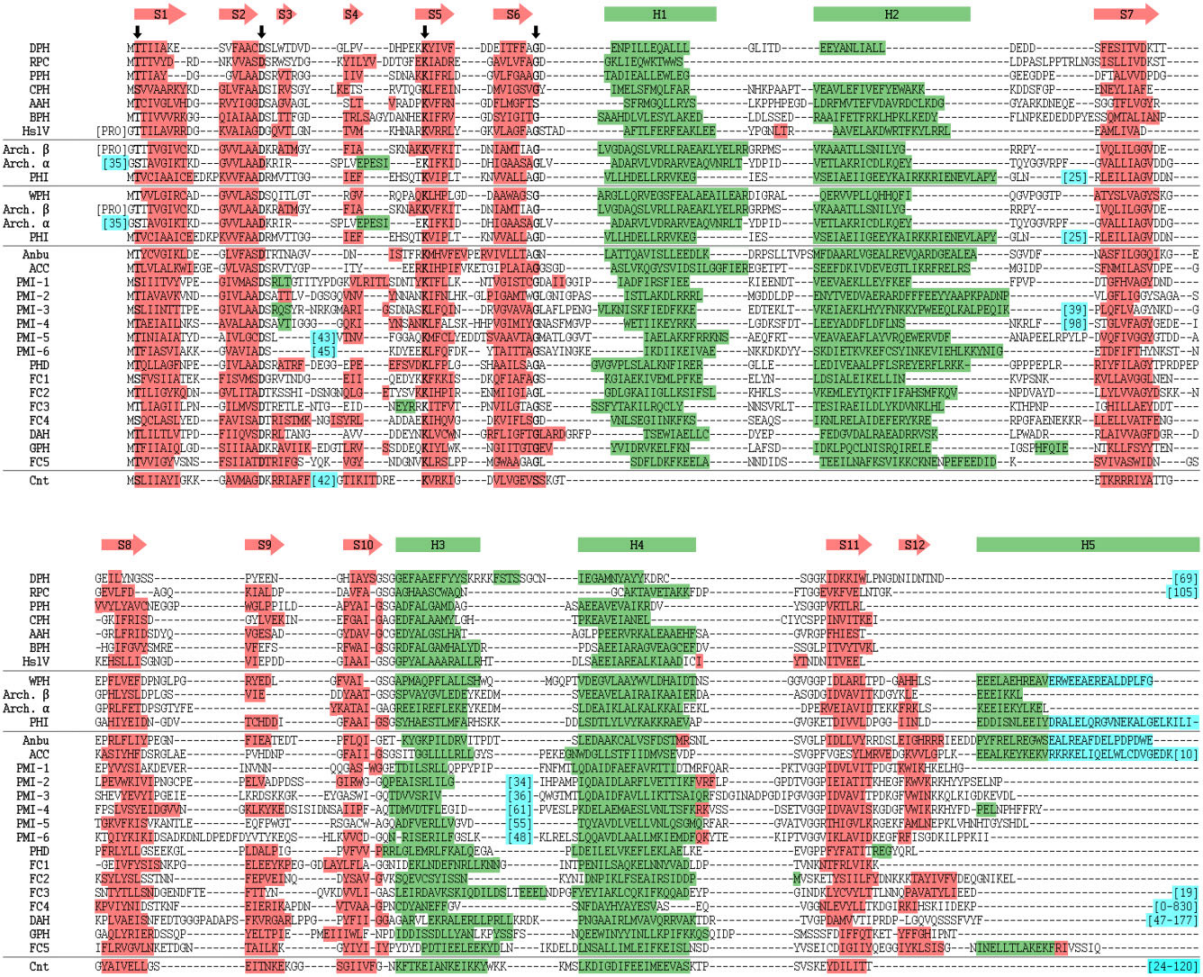
Structure-based sequence alignment of proteasome-like homologs. Shown is an alignment of consensus sequences with the secondary structure elements colored green (α-helices) and red (β-sheets). Insertions and N-/C-terminal extensions are shown in cyan and conserved residues are indicated by an arrow. Due to the extremely low sequence similarities, the alignment was primarily guided by the predicted structural models and the conservation of active site residues, and subsequent manual refinement. Connectase is abbreviated as Cnt. Comprehensive sequence alignments for each proteasome-like homolog can be found online at https://data.mendeley.com/drafts/t48yhff7hs/3

The alignment (Fig. 6) was calculated by feeding consensus sequences of proteasome homologs and the structures modeled by the tFold server (see above) to PROMALS3D (Pei *et al.*, 2008). The resulting alignment was refined manually to make it more comprehensible by reducing the number of gaps. In particular, long insertions were excluded, and specific secondary structure elements (e.g. helices) were realigned with MAFFT (Katoh and Standley, 2013) using a gap opening penalty of 3.5. Finally, the secondary structure information, as predicted by PROMALS3D, was plotted onto the alignment.

## 3 Results

### 3.1 Identification and cluster analysis of proteasome-like proteins

To investigate whether the diversity of the proteasome-like family is limited to or extends beyond its currently known members, we searched the non-redundant protein sequence (nr) database at NCBI for remotely homologous members using PSI-BLAST. The searches were seeded with the protein sequences of *Saccharomyces cerevisiae*, *Thermoplasma acidophilum*, and *Mycobacterium tuberculosis* proteasome subunits; *Methanosarcina mazei* connectase; *Pseudomonas aeruginosa* Anbu; *Haemophilus influenzae* HslV; and *Cupriavidus metallidurans* BPH. To detect hitherto unknown homologs, we inspected the non‐significant part of these PSI‐BLAST searches, i.e. matches with an E-value > 0.002, for the conservation of active site residues. To substantiate the membership of such low-scoring but possibly biologically meaningful matches in the proteasome-like family, further analyses were performed, including sequence clustering, structure prediction and sensitive sequence searches based on hidden Markov models (HMMs; see Section 2). Novel homologs identified in this manner were used as seeds for subsequent PSI-BLAST searches. By repeating this procedure iteratively, we detected a total of 22 novel subfamilies, which are related to the subunits of the 20S proteasome and which we named according to their taxonomic distribution and sequence characteristics. Many of these homologs only exhibit low pairwise sequence similarities to known proteasome-like homologs and therefore had remained undetected thus far. Nevertheless, as will be described in the following sections, these homologs are predicted to assume a proteasome-like fold and contain active site residues characteristic of the proteasome, making a solid case for their inclusion as new members of the proteasome-like family.

To visualize the sequence relationships between characterized proteasome-like family members and these novel homologs on a global level, we clustered them in CLANS *(*Frickey and Lupas, 2004; Fig. 3) based on their all-against-all BLAST+ *P*-values. CLANS represents protein sequences as points in a virtual two-dimensional space. In this space, points attract or repel each other based on their pairwise similarities, yielding a map in which related sequences form connected clusters, whereas unrelated ones gravitate to the periphery. The obtained map and a user guide are available for download at the Mendeley public repository (https://data.mendeley.com/datasets/t48yhff7hs/3) and can be opened with the CLANS program (https://www.eb.tuebingen.mpg.de/protein-evolution/software/clans/).

In the cluster map, proteasome α and β subunit sequences occupy a pivotal position, making strong connections to each other and to most other clusters. While the archaeal α and β subunits form the strongest connections to each other, the eukaryotic and bacterial α and β subunits are more divergent and radiate from the respective archaeal clusters, suggesting that the archaeal proteasome might represent the most original form of the proteasome that we can observe today. Furthermore, the ubiquitous occurrence of the proteasome in archaea and eukaryotes and deep-branching bacteria, primarily Actinobacteria, highlights the possible emergence and divergence of the proteasome from a shared ancestor that was already established at the time of the last universal common ancestor (LUCA).

The tightest connections to the proteasome clusters are made by HslV and Anbu clusters, and two clusters comprising novel archaeal and bacterial proteasome-like homologs, WPH (Widespread Proteasome Homolog) and PHI (Proteasome homolog with Helical Insertion). The remaining proteasome-like homologs are further removed from the proteasome clusters. Some of them share common sequence characteristics with HslV and BPH (group I), while others are more similar to WPH and Anbu (group II).

Group I clusters consist exclusively of bacterial sequences, with BPH occupying a central position. HslV and three novel proteasome-like homologs, AAH (α-proteobacterial and Actinobacterial proteasome Homolog), CPH (Cyanobacterial Proteasome Homolog) and PPH (Proteasome homolog of Phages and their Hosts), radiate from the BPH cluster. PPH forms connections to the more divergent RPC cluster (Related to PPH, with C-terminal extension), which is, in turn, connected to DPH (Divergent Proteasome Homolog).

Group II includes both archaeal and bacterial sequences, many of which make good connections to the aforementioned WPH cluster. Although WPH may present an evolutionary link between proteasome subunits and group II, we chose not to include it into this group, because it also shows high similarity to several group I proteins. WPH is connected to Anbu and a variety of novel clusters: (i) the PMI (Proteasome homolog with Multiple Insertions) variants, (ii) PHD (Proteasome Homolog of Deltaproteobacteria), (iii) ACC (Archaeal proteasome homolog with Coiled Coil) and (iv) FC1 (Firmicute Cluster 1). FC1 comprises a diverse group of proteins and is connected to other Firmicute clusters, FC2, FC3, FC4 and FC5 (Firmicute Cluster 2/3/4/5). These are, in turn, connected to GPH (Gammaproteobacterial Proteasome Homolog) and DAH (Distant Actinobacterial proteasome Homolog).

To obtain a systematic view of pairwise sequence relationships between proteasome-like homologs, we calculated profile HMMs for them and performed all-against-all comparisons using the sensitive sequence comparison method HHpred (Soding, 2005; Fig. 4). Like in the cluster map, proteasome β and WPH made the strongest connections to other homologs in these comparisons. Furthermore, these comparisons also showed that group I proteins are much more similar to the proteasome β subunit than the proteasome α subunit. In contrast, WPH and several group II proteins display high sequence similarity to both subunits. This similarity pattern likely reflects the evolutionary events that led to the emergence and divergence of group I and II proteins. A protein descended from the proteasome β subunit would be expected to be far more similar to β than to α subunits. On the other hand, a descendant of the proteasome precursor, i.e. the ancestral prototype that gave rise to proteasome α and β, would be expected to be similar to both proteasome α and β. This raises the possibility that while group I proteins, in the form of HslV, evolved from proteasome β subunits at the root of bacteria, WPH and group II proteins date back to the LUCA.

### 3.2 Phylogenetic distribution of proteasome-like proteins

Like Anbu, BPH, connectase and HslV, which are primarily found in prokaryotes, the novel proteasome-like homologs described in this study are also limited to prokaryotes and some phages (Table 1). Many of these proteins only have few representatives or show a narrow phylogenetic distribution, indicating either a less essential or a phylum-specific function for them. However, such proteins can still be of considerable biological interest, as demonstrated by the study of connectase, a protein ligase that occurs only in some methanogenic archaea (Fuchs *et al.*, 2021). We note that there are also some proteins with a higher number of representatives (e.g. AAH, PPH, PMI, FC1, GPH) and a broader phylogenetic distribution (e.g. WPH, PHI, PMI).

In general, we found no evidence of mutually exclusive proteasome-like homologs. Instead, the different homologs frequently co-occurred, indicating diverse or complementary functions. Group II proteasome-like homologs are found in bacteria and archaea, while group I occurs exclusively in bacteria and bacteriophages. A particularly interesting case is PPH, a proteasome-like homolog comprising just ∼140 residues, which occurs in various phages of the order Caudovirales and their bacterial hosts. Almost all bacterial PPH genes are flanked by other phage-derived genes, indicating that they were inserted there in the course of an infection. The only exceptions are species of the *Pseudoalteromonas and Mesorhizobium* genera, in which PPH is frequently found in context with other genes typical of bacteria. Thus, although PPH might also play a role in bacterial physiology, it can be considered the first true viral member of the proteasome-like family. Similar to PPH, AAH is also found in many phage genomes, and there is evidence that AAH had been inserted in genomes of the phyla Firmicutes and Bacteroidetes through phages. However, no evidence of their transmission through phages could be detected in Actinobacteria, where AAH is most abundant, and no Actinobacteria-specific phages that encode for AAH could be found. These observations show that phages played a central role in the distribution and evolution of PPH and AAH and may be responsible for the vast diversity of these proteins (Fig. 1).

### 3.3 Structural analysis of proteasome-like proteins

To analyze shared and unique sequence features of proteasome-like homologs, we explored the suitability of the best-performing, publicly available structure prediction servers from the recent Critical Assessment of Structure Prediction (CASP14) experiment, tFold (https://drug.ai.tencent.com), Robetta (Park *et al.*, 2018) and I-TASSER (Zhang, 2008). As a test, we used these servers to predict the structure of the highly divergent proteasome-like homolog connectase before depositing its experimental structure determined by us to the Protein Data Bank. The model yielded by the tFold server was impressively close to our experimental structure (Supplementary Fig. S1), and we, therefore, used it to predict the structure of the other proteasome-like homologs (Fig. 5). Although the obtained models still come with a certain level of inaccuracy, they are indeed our best guess for the structure of these proteins and are therefore valuable for visualizing their differences. We also used the predicted models to build a structure-based multiple sequence alignment of proteasome-like homologs (Fig. 6), as building one merely using sequence information is fraught with issues, owing to their extreme divergence.

The modeled structures show that all proteasome-like homologs share a conserved β-sheet core (S1—S11) that harbors the catalytic triad [Thr1/Ser1, Asp17, Lys33 in the proteasome (Huber *et al.*, 2016); Fig. 6] and the oxyanion hole (Gly47). Furthermore, most of them also contain two pairs of flanking helices (H1–H4). The sole exceptions are connectase, which does not encode for the first pair of helices, and PPH/RPC, which lacks the second helix (H2). Beyond the conserved core, various insertions or extensions are found in the individual representatives (cyan in Figs 5 and 6).

At the N-terminus, the catalytically inactive proteasome α subunits contain helical extensions that function as gate-keepers of the proteasome complex (Choi *et al.*, 2016). Furthermore, propeptides are found in most proteasome β and HslV subunits but not in any other proteasome-like homologs. Unlike the propeptides of other proteases, the proteasomal propeptides appear not to be required to prevent premature and unregulated proteolytic activity, as individual proteasome subunits without a propeptide are inactive and only become active in the context of the assembled complex (Seemuller *et al.*, 1996). Instead, they facilitate the assembly of the proteasome complex in bacteria and eukaryotes (Chen and Hochstrasser, 1996; Suppahia *et al.*, 2020), while currently having no known function in archaea.

A second insertion site is found between β strands 3 and 4 (S3/S4). This site is particularly interesting because it harbors the signature element of connectase (Fuchs *et al.*, 2021), a protein ligase that may prove useful in biotechnological applications. Here, a mobile, two-helical insertion covers the active site and contributes to the high substrate specificity of connectase. An insertion at this position is also found in PMI-5 and PMI-6. While the one in PMI-5 is predicted to be unstructured, the insertion in PMI-6 forms two helices reminiscent of the insertion in connectase and could potentially serve a similar purpose.

A third insertion site is located after the first two helices (H1/H2). In the proteasome, a loop, which lines the inner cavity and governs its chemical composition, is located at this position. This ‘pore loop’ is generally longer in the α subunits than in the β subunits and forms an additional layer that potentially regulates substrate access to the proteolytic core. This interpretation is also supported by the structures of the bacterial double-ring barrels HslV and BPH: While the pore loop is very short in HslV, which is regulated by the ‘gate-keeper’ HslU, it is much longer and highly acidic in BPH, which is apparently not regulated by an unfoldase. Furthermore, mutations at this site can lead to higher and unregulated proteolytic activity in HslV (Park *et al.*, 2013). Consequently, this site could also have regulatory functions in the other proteasome-like homologs. While many of them possess pore loops of different lengths, longer insertions are found in PHI, PMI-3 and PMI-4. If these insertions assume the orientation exhibited by the predicted models, they should prevent the assembly of ring structures, as they would protrude toward the central cavity and induce steric clashes. However, they do not make interactions to the core structure and could therefore also assume a different angle in the assembled complex, where they face outwards of the (hypothetical) ring, potentially forming a regulatory ‘cap’.

A fourth insertion is found between helices 3 and 4 (H3/H4) in PMI-2, PMI-3, PMI-4, PMI-5 and PMI-6. As these helices mediate the main contacts between two rings of proteasome β subunits, insertions at this position could prevent the assembly of double-ring layers.

Finally, the C-termini constitute the most divergent elements in proteasome-like homologs. Many proteasome subunits encode for a fifth helix (H5) at this position that stabilizes the double β-subunit layer. In Anbu, H5 is longer and forms coiled-coil interactions to the opposing subunit. Based on this observation, we have previously speculated that these components could have evolved early in the evolution of proteasome-like proteins to establish the double-ring structure (Fuchs *et al.*, 2017; Fuchs and Hartmann, 2019). Interestingly, such coiled-coil interactions are also predicted for the phylogenetically widespread homologs WPH, ACC and some PHI representatives.

Besides containing insertions and extensions, connectase, DAH, FC4, RPC and DGH also appear to contain additional C-terminally located domains. While the sequences of these hypothetical domains in RPC, DAH and DGH do not match known structures, the C-terminal domain in some connectase variants folds into a small β barrel and the C-terminal domains in FC4 show similarities to phage tail proteins. As the C-termini generally face away from the (assumed) ring-structures, such extensions could present potential interaction sites.

Taken together, all these elements account for a huge diversity and some of them appear to be incompatible with multi-layered ring structures, as formed by the proteasome, HslV or BPH. Therefore, it is not unlikely that the characterization of these new proteasome-like homologs will uncover novel, unprecedented quaternary structures.

## 4 Discussion

We have discovered a range of hitherto unknown proteasome-like homologs and analyzed their sequence relationships and characteristics. We have limited clues as yet about the potential function of these proteins. We found no genomic contexts or gene co-occurrence patterns suggestive of a specific function, nor could we obtain any publicly available microarray data for inferring their function. In particular, no co-occurring AAA+ interactors could be identified, raising the possibility that some of these homologs might function without specialized accessory factors or interact only with housekeeping genes. Support for this idea comes from experiments investigating oxidative stress, where the capped 26S proteasome is disassembled to the uncapped 20S proteasome, which then interacts with the housekeeping molecular chaperone Hsp70 for removal of oxidatively damaged proteins (Reeg *et al.*, 2016). In recent years, it has become increasingly evident that the proteasome not only functions in concert with AAA+ proteases in the well-known targeted protein degradation pathway but also has a physiological ATP-independent function in the clearance of unstructured and toxic proteins (Kumar Deshmukh *et al.*, 2019; Olshina *et al.*, 2020; Raynes *et al.*, 2016; Sahu *et al.*, 2019). It is possible that such functions are evolutionary more ancient and originated before a stable interaction with AAA+ proteins evolved. In agreement with this, the only known AAA+ interactor of HslV, HslU, does not belong to the same evolutionary clade as proteasomal AAA+ interactors (Ammelburg *et al.*, 2006) and uses a different interaction interface (Sousa *et al.*, 2000; Yu *et al.*, 2010), raising the possibility that these interactors were recruited independently by the established proteasome and HslV complexes.

The proteasome and some of its homologs appear to have emerged and radiated early in evolution. The proteasome itself occupies a pivotal position in the cluster map and is found in all domains of life. The observed distribution (Table 1) and sequence similarities (Figs 3 and 4) are hard to explain merely with horizontal gene transfer events, indicating that the proteasome instead emerged at the time of the LUCA and was subsequently lost in several bacterial branches. If we are correct about this assumption, proteasome subunits could have served as templates for the evolution of some of the other proteasome-like homologs. We have discussed this possibility based on the similarity patterns seen in the heatmap (Fig. 4) for HslV, which may present a link between bacterial proteasome β subunits and group I proteasome-like homologs. As all representatives of this group are limited to bacteria (and bacteriophages; Table 1), HslV could have evolved early in bacterial evolution and subsequently diversified into BPH, AAH, CPH and PPH. Compared to other proteasome-like family members, these homologs share a more compact subunit structure without insertions and without the C-terminal S12/H5 elements (Figs 5 and 6). The remaining group I homologs, PPH, RPC and DPH, are all exclusively found in proteobacteria (Table 1), suggesting that they represent more recent descendants.

**Table 1.** Relative abundance of proteasome-like homologs across all domains of life

Protein	KEGG orthologous cluster number	No. of seqs (nr90)	No. of seqs (nr70)	Proteobacteria	Firmi cutes	Actino bacteria	Bacteroi detes	PVC	Cyano bacteria	Other Bacteria	Euryar chaeota	Crenar chaeota	Other Archaea	Phages	Eukary ota
DPH	OC.1476421	37	28	X											
RPC	OC.1493145	79	58	X											
PPH	OC.1434257	324	219	X		X		X		X				X	
CPH	OC.1659476	27	17	X	X	X	X		X	X				X	
AAH	OC.661151	240	157	X	X	X	X		X	X				X	
BPH	OC.1456017	291	108	X	X		X	X	X	X					
HslV	OC.678827 - OC.678882	6072	576	X	X		X	X		X					X*
WPH	OC.1345157, OC.1226637	90	37	X		X		X		X	X	X	X		
Psm-β	OC.1176638, OC.1362555	>10000	1155	X		X		X		X	X	X	X		X
PHI	OC.1181028	53	52	X	X			X		X	X	X	X		
Anbu	OC.1323057	3087	595	X	X	X	X	X	X	X					
ACC	OC.1179207	17	15								X	X	X		
PMI-1	OC.651802	46	36	X	X		X	X		X	X				
PMI-2	OC.651795 − OC.651800 + OC.651804	103	80	X	X	X	X	X		X	X				
PMI-3	OC.651803	28	11	X						X	X				
PMI-4	OC.661505 − 661513	501	411	X	X	X	X	X	X	X	X			X	
PMI-5	OC.651801	19	12	X											
PMI-6	N/A	17	15	X			X			X					
PHD	OC.1959503	55	38	X	X					X					
FC1	OC.1697167, OC.1422923, OC.1423414, OC.1734992, OC.1428139	125	98		X										
FC2	OC.1712501	28	22		X										
FC3	OC.1702072	20	18		X										
FC4	OC.1268426	62	59		X										
DAH	OC.1345722, OC.350258, OC.1357815	77	68	X		X			X	X				X	
GPH	OC.1454157	159	97	X							X				
FC5	OC.1759113, OC.1735106	18	15		X	X									
Connectase	OC.1185479	111	66								X				

*Note*: Shown are the number of sequences for each protein in the non-redundant protein sequence database filtered for a maximum pairwise identity of 90% (nr90) and 70% (nr70), and their phylogenetic distribution. A relative overrepresentation of sequences is indicated by red crosses and black/gray crosses signify a somewhat lower or spurious protein occurrence. Note that proteins with few representatives will be found in only a small subset of the respective phyla (e.g. DPH is found in only a small number of proteobacteria, although marked by a red cross). The KEGG orthologous cluster numbers can be used to access a representative set of sequences for each protein (https://www.genome.jp/tools/oc/). Proteasome β subunits are abbreviated Psm-β; HslV is found in some organelles of endosymbiotic origin (*).

Compared to group I proteins, the evolutionary history of group II proteins is harder to reconstruct. Several of these proteins occur in both bacteria and archaea (Table 1), but their distribution appears to have been influenced by horizontal gene transfer events, as reflected by sequence similarity patterns in some instances. Nevertheless, some representatives could potentially date back to the time of the LUCA. A remarkable expansion is observed in firmicutes, which encode for five unique group II proteins. A further exceptional variation is observed in the PMI proteins, whose insertions account for very different structures, perhaps allowing these proteins to bind various interactors.

Taken together, the discovery of new proteasome-like homologs sheds a different light on the characteristics and evolution of the proteasome-like family. It appears that these proteins are widespread throughout all prokaryotic branches and exhibit remarkable diversity. This diversity is perhaps not entirely unanticipated: A comparison with other members of the Ntn-hydrolase clan, such as glutamine PRPP amidotransferase (Chen *et al.*, 1997), penicillin acylase (Suresh *et al.*, 1999) or phospholipase B (Lakomek *et al.*, 2009), reveals the tremendous structural and functional versatility of this clan (Supplementary Fig. S2). Many Ntn-hydrolases share no detectable sequence similarity, and, at first glance, their structures appear to show almost no resemblance. Each protein features numerous unique insertions, and the order of homologous elements on the polypeptide chain is sometimes shuffled or even split onto two polypeptide chains (Oinonen and Rouvinen, 2000). However, a closer inspection shows that they all share a common core fold and active site architecture. Given this considerable heterogeneity, it is not surprising that it was so far not possible to find a link between these Ntn hydrolases and proteasome-like proteins on the sequence level. Nevertheless, our study shows that with an ever-increasing number of sequenced genomes and more refined computational tools, we should be able to detect more and more diverse proteasome-like family members. It appears likely that many more representatives are still out there, and with their discovery, we may one day be able to understand the relationships between all Ntn hydrolase subfamilies.

## Funding

This work was supported by institutional funds from the Max Planck Society.

*Conflict of Interest*: none declared.

## Author contributions

A.C.D.F. discovered the novel proteasome homologs. A.C.D.F. and V.A. performed the bioinformatic analysis and wrote the paper. A.N.L. supervised the project.

## Supplementary Material

btab558_Supplementary_Data
