# Supplementary material for: An astonishing wealth of new proteasome homologs
Source: Bioinformatics. 2021 Jul 29;37(24):4694–703. doi: 10.1093/bioinformatics/btab558 (PMC8665760; doi:10.1093/bioinformatics/btab558)
Supplement: btab558_Supplementary_Data [file btab558_supplementary_data.docx]

# **Supplementary Data: An astonishing wealth of new proteasome homologs**

Adrian C.D. Fuchs, Vikram Alva, Andrei N. Lupas*

Department of Protein Evolution, Max Planck Institute for Developmental Biology, 72076 Tübingen, Germany

^*^ To whom correspondence should be addressed.

**Contact:** [andrei.lupas@tuebingen.mpg.de](mailto:xxxxx@xxxx.xxx); Phone: +49 7071 601341


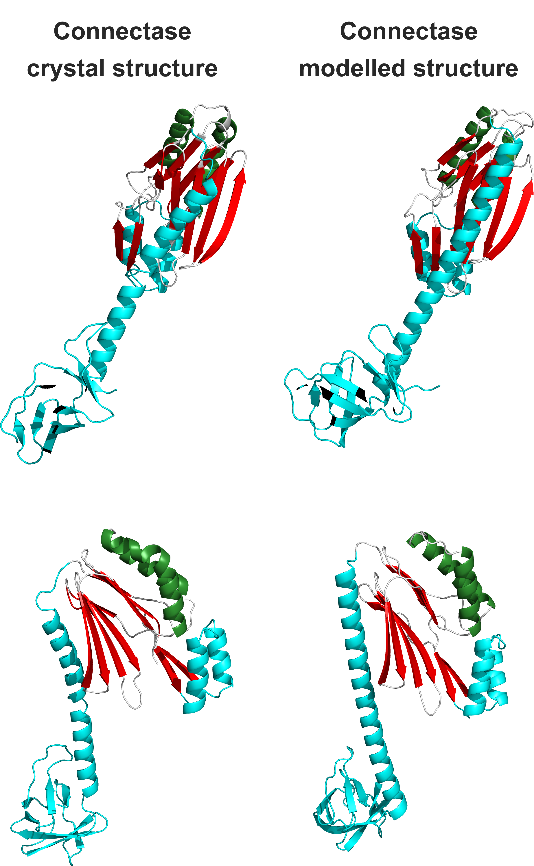


**Fig. S1: Novel structure prediction methods produce useful models for divergent proteasome homologs.**

Shown is the Connectase crystal structure as well as a prediction of the Connectase structure in the same orientation as in Fig. 5 (top) and in a 90° rotated orientation (bottom). Connectase diverges from other proteasome homologs in an insertion of two helices (cyan) and in the deletion of two other helices (H1/H2 in Fig. 6). Common template-assisted methods typically aligned these divergent elements, resulting in entirely wrong structural predictions. By contrast, the template-free tFold server predicted a structure that came remarkably close to our experimental structure and mostly diverged in the orientation of the flexible C-terminal elements.


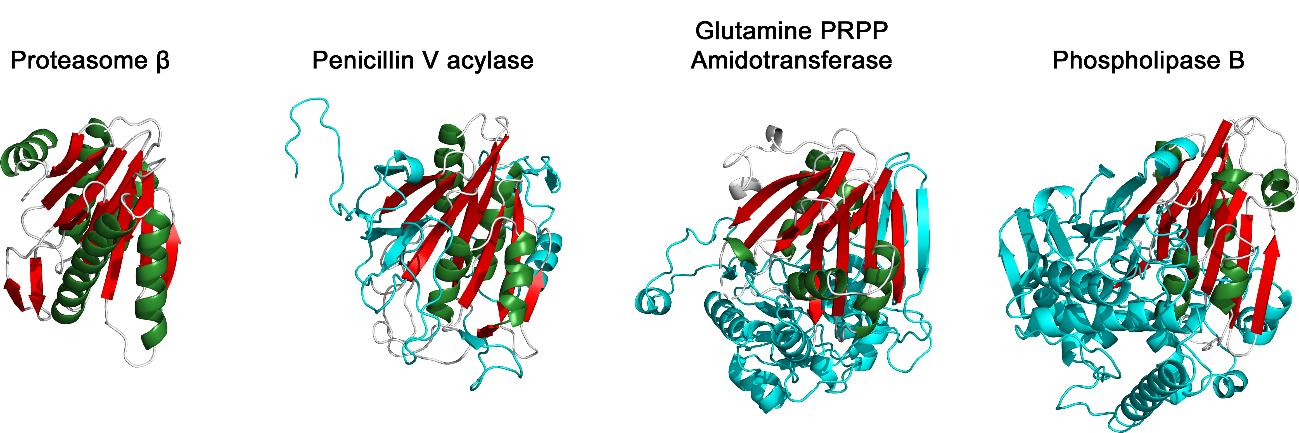


**Fig. S2: Ntn hydrolases are structurally diverse.**

Shown are the subunits of four Ntn hydrolases: the β subunit of the tetradecameric proteasome (PDB code 1PMA), subunits of the tetrameric Penicillin V acylase (3PVA) and Glutamine PRPP amidotransferase (1AO0) complexes, and monomeric Phospholipase B (3FBX). The Ntn core domain is colored in red (sheets) and green (helices) and insertions that are not found in the proteasome β subunit are shown in cyan. Such insertions are abundant throughout the Ntn hydrolase superfamily at various positions of the fold. In case of Glutamine PRPP amidotransferase, the structural elements in the Ntn core domain are arranged in a different order compared to the other structures.
